# Development of a Novel Steam Distillation TBA Test for the Determination of Lipid Oxidation in Meat Products

**DOI:** 10.3390/foods12020359

**Published:** 2023-01-12

**Authors:** Eugenios Katsanidis, Konstantina Zampouni

**Affiliations:** Department of Food Science and Technology, School of Agriculture, Faculty of Agriculture, Forestry and Natural Environment, Aristotle University of Thessaloniki, 54124 Thessaloniki, Greece

**Keywords:** TBA test, lipid oxidation, meat, steam distillation

## Abstract

The 2-thiobarbituric acid (TBA) method has been used for the spectrophotometric determination of secondary lipid oxidation products, such as malonaldehyde (MA), due to its good correlation with sensorial perception of lipid oxidation. Other approaches have been proposed over time. Direct distillation can result in artificially increased MA concentrations due to intense heating. Extraction is a milder and faster method, but it suffers from false color development in the presence of sugars or other compounds. A novel approach using steam distillation for the recovery of MA was developed. Validation and optimization studies were conducted, aiming to maximize MA recovery from various meat product samples by adjusting the steam distillation parameters. For the optimal MA recovery, 10 g of the sample, 25 mL of H_2_O, and 5 mL of 2 N HCl were used. The sample was distilled using a stream of water vapor until 50 mL of the distillate was collected in less than 3 min. Subsequently, 5 mL of the distillate was reacted with 5 mL of 0.02 M TBA, and the absorbance was measured at 532 nm. MA recovery was 61.8%. Experimentation with varying nitrite levels suggested that the addition of sulfanilamide is necessary when NaNO_2_ is more than 50 mg MA/kg. The proposed method is fast, milder than direct distillation, and eliminates the issue of TBA interacting with sugars and other compounds.

## 1. Introduction

Lipid oxidation is one of the most important mechanisms of food deterioration [1,2]. Furthermore, the formation of free radicals can have adverse effects on human health, and it has been correlated with an increased risk of coronary disease and various forms of cancer [3,4,5]. One of the classical methods for assessing lipid oxidation in food products is the TBA test, where the secondary oxidation products of the food lipids, such as malonaldehyde (MA), are reacted with 2-thiobarbituric acid to produce a chromophore that absorbs at 532 nm [6,7]. The term TBA-reactive substances (TBARS) has been used by many researchers, reflecting the fact that not only MA but several aldehydes and other oxidation products can react with 2-thiobarbituric acid. Therefore, even though the results of the TBA test are typically expressed as milligrams MA per kilogram, they reflect the overall oxidation status of the food product encompassing many secondary lipid oxidation products [2]. The TBA test is useful due to its strong correlation with sensorial perception of lipid oxidation in food, and it has been used in different variations over the last 60 years. It remains one of the most commonly used assays for measuring lipid oxidation, especially in meat and meat products. However, there are several issues regarding the implementation of the TBA test that have led to various approaches and analytical techniques regarding (a) the process of isolating MA from the food sample, (b) the optimal conditions for the reaction of TBA with MA, and (c) the elimination of potential interferences from the reaction of 2-thiobarbituric acid with other compounds present in the food sample.

The method first reported by Tarladgis et al. [8,9] involves the direct distillation of the acidified food sample for the recovery of MA and the consequent reaction of MA with 2-thiobarbituric acid at 100 °C for 35 min. A significant issue with this method has been that the intense heating of the food sample during distillation promotes further sample oxidation and production of MA, thereby artificially increasing the results of the TBA test. Many variations in the original method have been proposed by different researchers [2,10] to address the aforementioned issue. In 1987, Salih et al. [11] proposed the isolation of MA from chicken meat through an extraction process instead of distillation, thus avoiding sample heating. While this method is appropriate for pure meat samples, it can extract colored substances present in the food sample or produce color artifacts in the presence of compounds containing aldehyde or ketone groups, such as sugars, thus increasing the TBA number and rendering it unsuitable for the measurement of lipid oxidation in various meat products [2,12].

Another issue that has been studied is the interaction of MA with nitrites, typically present in cured meat products. Residual nitrites can react with MA, making it unavailable to react with TBA, thus resulting in lower TBA numbers [13]. The addition of sulfanilamide (SA) has been proposed to prevent NaNO_2_ from reacting with MA [14,15]. However, the effectiveness of this practice has also been questioned because SA was reported to react with MA when there is no residual nitrite in the food sample [13].

The objective of this study was to develop and validate an alternative methodology to perform the TBA test by used steam distillation, which provides a faster and milder heating method designed to minimize the artificial formation of MA and avoid extracting compounds that can interfere and artificially increase the TBA number by reacting with 2-thiobarbituric acid. Furthermore, the usefulness of the addition of SA in the presence of nitrites was evaluated for the proposed steam distillation method.

## 2. Materials and Methods

Fresh ground beef, chicken breast meat, sardines, and sugar were purchased from a local grocery store in Thessaloniki, Greece, and were used as substrates for measuring lipid oxidation and for the comparison among the different TBA test methodologies. The extract from olive leaves was a kind donation from the OliveBoost Company (Athens, Greece). TBA (Alfa Aesar, Karlsruhe, Germany), HCl (Merk KGaA, Darmstadt, Germany), perchloric acid (Chem-Lab NV, Zedelgem, Belgium), silicon antifoam solution (Sigma-Aldrich, St. Louis, MO, USA), 1,1,3,3 tetra ethoxypropane (TEP) (TCI, Tokyo, Japan), butylated hydroxyanisole (J&K Scientific, Pforzheim, Germany), and sulfanilamide (SA) (Merk KGaA, Darmstadt, Germany) were used as reagents for the various TBA tests.

The TBA test developed by Tarladgis et al. [8] was used as a reference point for the steam distillation method development. This method is based on homogenizing 10 g of food sample with 97.5 mL of H_2_O, adding 2.5 mL of 4 N HCl, and 3–4 drops of silicon antifoam to prevent excessive foaming. We distilled the mixture until 50 mL of distillate was collected. Then, 5 mL of the distillate was reacted with 5 mL of 0.02 M TBA in a 98 °C water bath for 35 min. After cooling, the absorbance of the mixture was measured at 532 nm.

For the steam distillation method development, 10 g of the food sample was also used. The amounts of H_2_O used for sample homogenization and collected distillate were varied to optimize MA recovery from the process. The experimental plan included homogenization of the food samples with 30, 40, 50, or 100 mL of extraction solution (H_2_O and HCl) and, after steam distillation, collecting 25 or 50 mL of the distillate.

The final test procedure for the steam distillation TBA test involved homogenizing 10 g of the food sample with 25 mL of H_2_O for 1–2 min using an Ultra Turrax T18 homogenizer (IKA, Staufen, Germany) at 14,000 rpm. Next, the homogenate was transferred into a small distillation flask, and 5 mL of 2 N HCl and 3–4 drops of silicone antifoaming solution were added. The acidified samples were steam-distilled on a distillation unit (UDK 127, VELP Scientifica, Monza, Italy) until 50 mL of the distillate was collected in approximately 3 min. A 5 mL aliquot of the distillate was transferred into a test tube, and 5 mL of 0.02 M TBA was added. All samples were heated in a 98 °C water bath for 35 min and then cooled with cold tap water. Absorbance measurements were made using a Shimadzu UV-1700 spectrophotometer (Shimadzu Europe GmbH, Duisburg, Germany) at 532 nm against a blank containing 5 mL of deionized water instead of the distillate. The results were converted to TBA numbers (mg MA/kg sample) using a conversion factor calculated from the standard curve and the recovery studies.

For the recovery studies, appropriate quantities of MA were mixed with different food samples (e.g., ground beef and ground chicken meat), and the samples were subjected to the steam distillation procedure, as described above. Recovery (%) was calculated using Equation (1):

(1) $$\%\;\mathrm{Recovery}=100\times \frac{Abs_{\left(\mathrm{sample}+\mathrm{MA}\right)}-Abs_{\left(\mathrm{sample}\right)\;}}{Abs_{\left(\mathrm{MA}\right)\;}}\;$$

where $Abs_{\left(\mathrm{sample}+\mathrm{MA}\right)}$ is the absorbance of the sample with the added MA, $Abs_{\left(\mathrm{sample}\right)}$ is the absorbance of the plain sample, and $Abs_{\left(\mathrm{MA}\right)}$ is the absorbance of the added MA.

For the preparation of the standard curve, 70–80 mg of 1,1,3,3 tetraethoxypropane (TEP) was accurately weighed, 10 mL 0.1 Ν HCl was added, and the solution was heated at 65 °C for 5 min so that TEP would be hydrolyzed into MA (1 mole TEP produces 1 mole MA). After rapid cooling, the mixture was brought to 100 mL with H_2_O (MA stock solution). Next, appropriate dilutions of the MA stock solution, ranging from 1.6 × 10^−8^ to 1.6 × 10^−7^ moles MA/5 mL, were reacted with 5 mL of 0.02 M TBA, and the absorbance was measured at 532 nm, as described above. The conversion of absorbance values to TBA numbers, taking into account the percent recovery of MA, was carried out according to [8].

The extraction TBA test [11] was used as the reference method for comparison with the steam distillation method. For the extraction method, 10 g of the food sample was homogenized with 35 mL of perchloric acid 0.4 N, 5 mL of H_2_O, and 1 mL of BHA 0.35 M in ethanol as an antioxidant to prevent further MA production. The homogenate was filtered (Whatman 2, GE Healthcare, Buckinghamshire, UK), and 5 mL of the filtrate was reacted with 5 mL of 0.02 M TBA. The absorbance was recorded as previously described. Ground beef, sardine fillets, ground beef with 2% sugar, and ground beef with 2% olive leaves extract were analyzed with both TBA methods.

To evaluate the necessity for adding sulfanilamide (SA) when nitrites are present in a sample, a solution with varying amounts of NaNO_2_ was added to ground beef so that the final samples would contain 0, 50, 100, or 200 mg of NaNO_2_/kg. We added 2 mL of a 0.5% solution of SA in 20% HCl in the distillation flask with the samples. SA has been reported to react with NaNO_2_ and prevent its reaction with MA [13]. For the assessment of the usefulness of SA addition, the percent recovery of MA from the samples with and without the addition of SA was measured, as previously described.

All reported results are the means of 3 replications. The comparison of the means was performed using ANOVA and the least squares means Student’s *t*-test (JMP v. 13.2, SAS, Cary, NC, USA).

## 3. Results

### 3.1. Optimization of the Steam Distillation Parameters

For the development of the steam distillation procedure, various quantities of extraction solution (H_2_O and HCl) were evaluated. Additionally, the volume of the collected distillate was varied to maximize the percent recovery of MA. Table 1 presents the percent MA recovery measured when the different steam distillation conditions were applied for the procedure.

In steam distillation, part of the steam injected into the flask with the sample is condensed, especially during the initial stages of the procedure, when the sample temperature is still low. Because of this, the quantity of H_2_O evaporated and distilled from the sample is constantly replenished by the condensed steam. Therefore, in contrast with the classical distillation method developed by Tarladgis et al. [8] or the method proposed by Sørensen and Jørgensen [16], an initial large quantity of extraction solution in the flask is not necessary for efficient sample distillation. Large quantities of extraction solution resulted in lower recoveries, indicating a less efficient distillation of MA from the sample. The highest recovery was obtained when 30 mL of extraction solution was used (25 mL of H_2_O and 5 mL of 2 N HCl). Extraction volumes lower than 30 mL were insufficient to adequately homogenize the food sample and were not evaluated.

MA is an aldehyde that is expected to distill in the early stages of the steam distillation procedure. In the classical distillation procedure, 50 mL of the distillate is typically collected. In our study, two distillate volumes (25 and 50 mL) were evaluated to shorten the distillation duration, thus minimizing the heating step that could artificially increase MA levels due to heat-induced lipid oxidation. The results in Table 1 indicate that the collection of 50 mL of the distillate is necessary for adequate recovery of MA and agree with previously published studies [8,13]. Overall, approximately 3 min of steam distillation was required to collect 50 mL of the distillate, a significantly shorter heating time than the conventional distillation process.

### 3.2. Standard Curve and Validation

A coefficient (K) was calculated for the conversion of absorbance to TBA number, as shown in Equation (2). The TBA number is expressed as milligrams of MA per kilogram so that results can be compared with the results of previously published studies.

(2) TBA (mg MA/kg)=(Absorbance)×(K)

**Figure 1:**
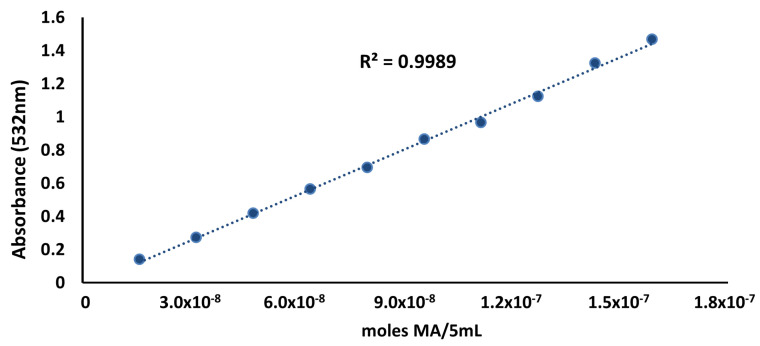
Standard curve of malonaldehyde for the steam distillation TBA method.

A very good fit of the standard curve data was obtained, as shown in Figure 1. The linear regression coefficient of the data presented in Figure 1 was 1.08 × 10^−7^ (moles MA/5 mL)/absorbance. This value was used for calculating the coefficient K according to Equation (3), as proposed by [8].

(3) K=moles MA5 mLAbsorbance×(MW of MA)×10−7sample wt×100% recovery

**Figure 2:**
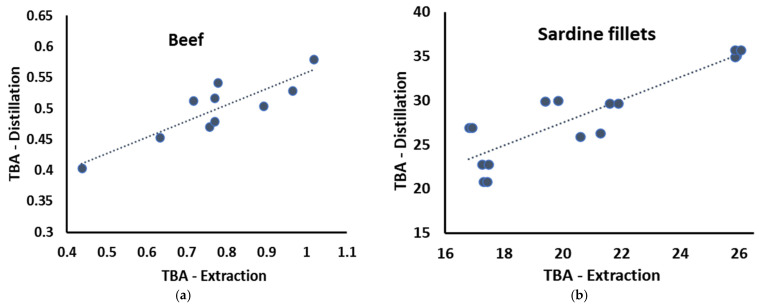
Correlation between the steam distillation and the modified extraction TBA method in different food samples. All TBA values in mg MA/kg. (**a**) ground beef; (**b**) sardine fillets.

The lipid oxidation of various food samples was measured using steam distillation. The results were compared with those of the modified extraction method proposed by [11] to assess the validity of the steam distillation method. In Figure 2, the correlation between the TBA values obtained by the two methods is presented for ground beef and sardine fillets stored for up to 4 days at 4 °C. In both cases, a highly significant correlation was observed. Specifically, for the ground beef samples, the Pearson correlation coefficient was 0.872 (*p* = 0.001) and 0.892 (*p* < 0.001) for the sardine fillets. These results suggested that the steam distillation method is suitable for measuring lipid oxidation in meat and fish samples with varying levels of lipid oxidation. It should be noted that even though a very high correlation between the two methods was established, the MA concentrations in beef samples estimated by the steam distillation method did not increase at the same rate as those estimated by the extraction method. A possible explanation could be that at such low MA concentrations, the different limitations of each method (heating vs. extracting unwanted compounds) impacted the results in a dissimilar manner. This observation should be considered when interpreting the results of the samples with low oxidation levels. The differences observed in the two food samples (beef vs. sardine fillets) could be attributed to the different food sample matrices and the differences in the two methods (heating vs. not heating). Specifically, we hypothesized that in the case of the beef samples analyzed with the extraction method, other compounds extracted from the beef tissue (i.e., nucleotides, etc.) also reacted with the TBA reagent and produced slightly higher values than the steam distillation method. This increase was measurable because lipid oxidation levels were low during the 4 days of storage due to the very-low-fat content of the beef tissue and the more saturated fatty acid profile. In the sardine samples, where the fat content was higher and the fatty acids were much more unsaturated and susceptible to oxidation, the effect of the heating (in the steam distillation method) resulted in increased MA values compared with the extraction method. However, the two TBA methods correlated very well for both food samples and could be used to assess lipid oxidation.

In several meat products and preparations, sugars are added for flavor and color development (e.g., honey-cured ham). Sugars have functional groups that can react with TBA, producing a chromophore that can absorb at the same range as the MA–TBA complex [2,12]. The application of naturally derived bioactive compounds with antioxidant or antimicrobial activity is gaining momentum in meat products [17,18,19]. However, many have functional groups or carry natural colorants that may also interfere with the TBA test.

To further test the suitability of the proposed steam distillation TBA method for meat products with different added ingredients, ground beef samples were formulated with 2% sugar or 2% olive leaves extract, stored for 4 days at 4 °C, and the lipid oxidation was measured with the steam distillation and the modified extraction methods. Olive leaf extract is a novel natural antioxidant from olive leaves that is rich in polyphenols (>32,000 mg/kg, unpublished data). Olive leaf extract has good antioxidant properties and an olive-green color that can affect spectrophotometer readings.

**Figure 3:**
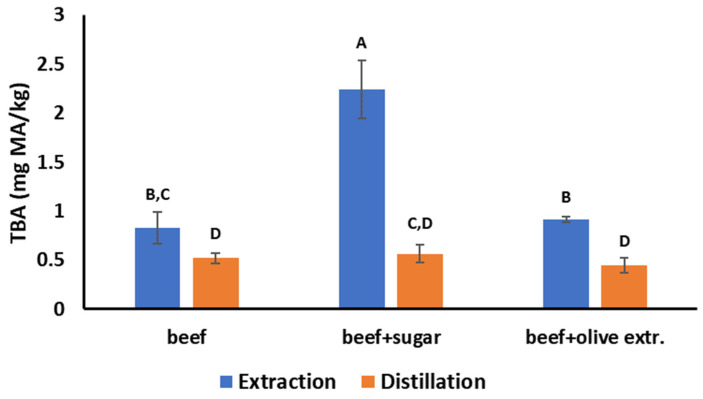
Comparison of the steam distillation and the modified extraction TBA method in plain ground beef, in ground beef with 2% added sugar, and in ground beef with 2% added olive extract. Different letters indicate statistically significant differences (*p* < 0.05) among the six treatments.

The steam distillation TBA values of the ground beef samples were not affected (*p* > 0.05) when 2% sugar was added because sugar is not volatile and does not evaporate into the distillate. In contrast, the values of the modified extraction TBA method were much higher (*p* < 0.05) in the presence of sugar because sugar was extracted in the filtrate and reacted with the TBA, falsely increasing the TBA number of ground beef (Figure 3). When 2% olive leaf extract was added to ground beef, the steam distillation TBA values slightly decreased because of the antioxidant action of the olive leaves extract, but this decrease was not statistically significant (*p* > 0.05). In the case of the modified extraction TBA method, the TBA numbers slightly increased (Figure 3), probably due to the interference of the color of the extract on the spectrophotometer readings, but this increase was not statistically significant (*p* > 0.05). Overall, the steam distillation method can produce consistent results and is not affected by the presence of other compounds that can interfere with spectrophotometric readings.

**Figure 4:**
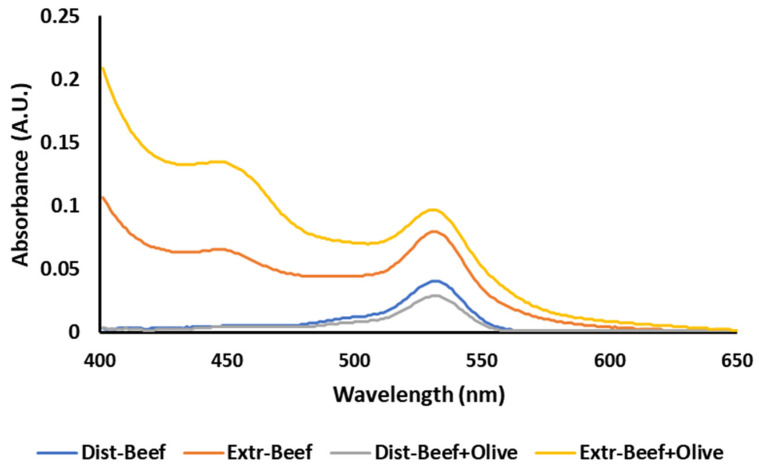
Absorbance spectra of the steam distillation (Dist) and the modified extraction (Extr) TBA method in plain ground beef and ground beef with 2% added olive leaf extract.

These results could be further elucidated by examining the absorption spectra of the two methods. The steam distillation method was not affected by interferences and produced a simple spectrum with one peak at 532 nm (Figure 4) and a flat baseline. The modified extraction method produced more complex spectra, with an ascending baseline and a second peak at around 450 nm (Figure 4) due to the presence of other colored compounds present in the extract.

### 3.3. Sulfanilamide and Nitrite

The presence of nitrites in cured meat products can affect the results of the TBA test due to the reaction of NaNO_2_ with MA. This nitrosation of MA occurs during sample homogenization and reduces the quantity of free MA that can react with the TBA reagent, resulting in lower TBA numbers. Sulfanilamide (SA) can react with residual nitrites present in cured meats, thus preventing the reaction of nitrites with MA [14,15]. However, residual SA that has not reacted with nitrites can also react with MA, adversely affecting the measurement of lipid oxidation [13].

**Figure 5:**
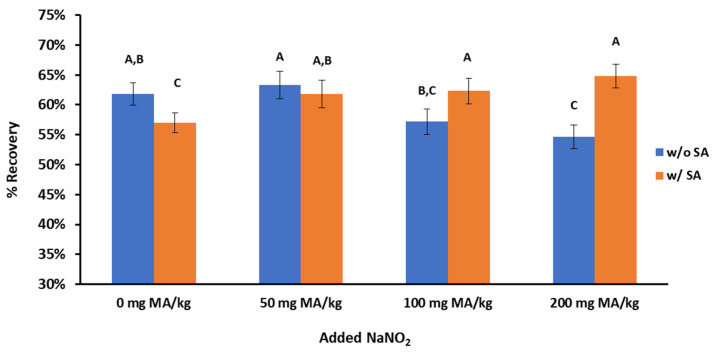
Recovery (%) of malonaldehyde for the steam distillation TBA method in the presence of NaNO_2_ and sulfanilamide (SA). Different letters indicate statistically significant differences (*p* < 0.05) among the eight treatments.

The usefulness of adding SA in the steam distillation method was assessed in ground beef samples containing increasing levels of nitrites. The percent recovery of MA was measured in ground beef samples containing 0, 50, 100, or 200 mg of NaNO_2_/kg. The addition of SA in ground beef samples with no added nitrites reduced the MA recovery (*p* < 0.05), supporting the hypothesis that the available SA can react with MA and decrease the percent recovery (Figure 5). When 50 mg of NaNO_2_/kg was added to ground beef, the MA recovery was not statistically different between samples with and without the addition of SA. Furthermore, for the samples with 0 and 50 mg of NaNO_2_/kg and no added SA, the percent recovery was very similar, indicating that adding SA is unnecessary for up to 50 mg of NaNO_2_/kg. For higher levels of nitrites (100 and 200 mg of NaNO_2_/kg), the percent recovery of MA decreased significantly (*p* < 0.05) without SA, indicating that the residual nitrites reacted with MA and did not allow for the complete quantification of MA. It should be noted that the percent recovery of MA did not statistically differ (*p* > 0.05) for samples with 50–200 mg of NaNO_2_/kg in the presence of SA, indicating that SA should be added in cured meat products that are typically formulated with 150 mg of NaNO_2_/kg.

## 4. Discussion

Various TBA test methods have been developed for measuring lipid oxidation in food samples. The modified extraction method [11] offers the advantage of being a rapid and mild process that does not involve heating of the sample during analysis, thus avoiding increased TBA numbers due to heat-induced MA formation. However, the extraction method can produce excessively high TBA values in the presence of colored compounds or other substances that can react with TBA [2,12]. The conventional distillation method [8], on the other hand, avoids the aforementioned issues of interference, but the prolonged heating and the high temperatures encountered in the distillation vessel can promote lipid oxidation during distillation. Specifically, in the classic distillation method [8], much more time (10–15 min) is required to heat 100 mL of solution (H_2_O and HCl) to boiling temperatures and collect 50 mL of the distillate, whereas in the steam distillation method, only 30 mL of solution is heated by steam injection, and this process lasts only 3 min. Additionally, in classical distillation, the heating element temperature is more than 200 °C, resulting in the formation of a “burnt crust” inside the distillation flask due to the high temperatures. In steam distillation, this phenomenon is not observed. So, overall, the steam distillation method is considered a milder heating process and results in less heat-induced degradation of the sample and less artificial MA formation. Moreover, the distillation method is generally considered more sensitive and appropriate for high-fat samples than the extraction methods [20].

## 5. Conclusions

A steam distillation method was developed and validated in various meat and fish products. The steam distillation method correlates very well with the modified extraction method for pure meat and fish samples. Moreover, the steam distillation method is not affected by possible interference by other compounds or colored ingredients. The addition of SA during sample homogenization is recommended when nitrites are present in the food sample. Steam distillation is a much faster procedure that minimizes sample heating compared with the conventional distillation process, thus avoiding further heat-induced lipid oxidation of the sample.

## Figures and Tables

**Table 1:** Recovery of MA under varying process parameters during steam distillation.

Extraction Solution Volume	25 mL Distillate	50 mL Distillate
30 mL	52.3% ^b^	61.8% ^a^
40 mL	41.5% ^c^	50.8% ^b^
50 mL	32.1% ^d^	50.3% ^b^
100 mL	30.4% ^d^	37.1% ^c^

^a–d^ Recoveries with different superscripts are statistically different (*p* < 0.05).

## Data Availability

The data presented in this study are available on request from the corresponding author.
